# Endophytic Fungi as Potential Biological Control Agents against Grapevine Trunk Diseases in Alentejo Region

**DOI:** 10.3390/biology9120420

**Published:** 2020-11-26

**Authors:** Angela Billar de Almeida, Jonathan Concas, Maria Doroteia Campos, Patrick Materatski, Carla Varanda, Mariana Patanita, Sergio Murolo, Gianfranco Romanazzi, Maria do Rosário Félix

**Affiliations:** 1 MED—Mediterranean Institute for Agriculture, Environment and Development, Instituto de Investigação e Formação Avançada (IIFA), Universidade de Évora, Polo da Mitra, Ap. 94, 7006-554 Évora, Portugal; gebillar@gmail.com (A.B.d.A.); pmateratski@uevora.pt (P.M.); carlavaranda@uevora.pt (C.V.); mpatanita@uevora.pt (M.P.); 2 Dipartimento di Scienze Agrarie, Alimentari ed Ambientali, Università Politecnica delle Marche, Via Brecce Bianche, 60131 Ancona, Italy; john.concas@gmail.com (J.C.); s.murolo@staff.univpm.it (S.M.); g.romanazzi@staff.univpm.it (G.R.); 3 MED—Mediterranean Institute for Agriculture, Environment and Development & Departamento de Fitotecnia, Escola de Ciências e Tecnologia, Universidade de Évora, Pólo da Mitra, Ap. 94, 7006-554 Évora, Portugal; mrff@uevora.pt

**Keywords:** grapevine trunk diseases, *Vitis vinifera*, molecular identification, endophytic fungi, antagonism tests

## Abstract

**Simple Summary:**

Grapevine trunk diseases are the most widespread fungal diseases, affecting grapevines in all the major growing regions of the world, and their complete eradication is still not possible. Aiming to search alternatives to avoid the spread and high incidence of these diseases, the present work identified in vineyards within the Alentejo region the grapevine fungal community and among it the fungi responsible for those diseases. Grapevine fungal community showed a wide variety of fungi, nine of them previously described as grapevine trunk diseases-associated fungi. Almost all these fungi were detected in symptomatic and asymptomatic plants, which shows the importance of investigating the interactions of fungal communities and confirms the need for early diagnosis of these diseases. The potential of endophytic fungi as bio-control agents was tested against grapevine trunk diseases-associated fungi. These tests were performed among identified endophytes and grapevine trunk diseases phytopathogenic fungi, and all the endophyte fungi showed potential as biocontrol agents. Our findings suggest that endophytes are promising candidates for their use in biological control due to their antagonistic activity against the mycelia growth of grapevine trunk diseases-associated fungi.

**Abstract:**

Grapevine trunk diseases (GTDs) are the most widespread fungal diseases, affecting grapevines in all the major growing regions of the world, and their complete eradication is still not possible. Aiming to search alternatives to avoid the spread and high incidence of these diseases, the present work intended to molecularly identify the grapevine endophytic community, the phytopathogenic fungi associated with GTDs in vineyards within the Alentejo region, and to test potential antagonist microorganisms as biological control candidates against GTDs-associated fungi. Grapevine endophytic community showed a wide variety of fungi in GTDs’ asymptomatic and symptomatic plants, nine of them previously described as GTDs-associated fungi. GTDs prevalent fungi identified in symptomatic plants were *Diaporthe* sp., *Neofusicoccum* sp., and *H. viticola*. Almost all these fungi were also detected in asymptomatic plants, which shows the importance of investigating the interactions of fungal communities and confirms the need for early diagnosis of these diseases. Direct inhibition antagonism tests were performed among identified endophytes and GTDs phytopathogenic fungi, and all the endophyte fungi showed potential as biocontrol agents. Our findings suggest that endophytes are promising candidates for their use in biological control due to their antagonistic activity against the mycelia growth of some GTDs-associated fungi.

## 1. Introduction

Grapevine (*Vitis vinifera* L. and *Vitis* spp.) belongs to the *Vitaceae* family and is one of the most economically important woody perennial fruit crops in the world [1]. Nowadays, Portugal is among the twelve major wine producers in the world with a production of 6.1 million hl in 2018 [2] and with the wine consumption growing in the last years. Alentejo (south Portugal) is the second-largest wine producer region of Portugal [3].

Grapevine species are constantly affected by fungal diseases, which cause important economic losses. Grapevine trunk diseases (GTDs) are the most important and destructive fungal diseases, affecting grapevines in all the major growing regions of the world [1,4,5]. These destructive diseases cause important damages every year with the need for replacement of dead grapevines worldwide and are the major causes of grapevine decline, especially in European countries [6,7,8]. GTDs are caused by wood-inhabiting fungi that cause the death of the spurs, canes, and/or cordons in mature and young plants, which impact grape and wine economic production, reducing productivity, quality, and longevity of the vineyards [7,9,10,11,12,13,14].

GTDs are related to 133 fungal species belonging to 34 different genera and nine different families, with similar life cycles and epidemiology [1,4]. The genera associated with GTDs mostly found worldwide are *Botryosphaeria*, *Diplodia*, *Lasiodiplodia*, *Fusicoccum*, *Neofusicoccum*, *Dothiorella*, *Phomopsis*, *Diaporthe*, *Eutypa*, *Eutypella*, *Diatrypella*, *Diatrype*, *Cryptovalsa*, *Cylindrocarpon*, *Phaeomoniella*, *Fomitiporia*, *Phaeoacremonium*, and *Greeneria* [5,15,16]. Grapevines can be infected by different pathogens over the years due to the multiple infection opportunities, and each plant can be affected by one or more GTDs at the same time [1,17].

GTDs-associated fungi live in and colonize the wood of the perennial organs, impeding the water transport in plants by clogging the xylem vessels and, consequently, decrease the adsorption capacity of water and nutrients [18,19,20]. However, their effect can be undetectable for years due to the slow growth of the pathogens in the vascular tissues, which enables the visibility of symptoms on the canopy [9]. In general, at the time of the appearance of leaf symptoms, the disease has already developed to a severe situation, which can lead, in some cases, to the death of a cordon or of the entire plant [21]. Nevertheless, some plants may remain with leaf symptoms for many years, reaching apoplexy after a heat stress situation [8]. Those fungi may live as endophytes, asymptomatically, during part of their life cycle and, at some point, modify their behavior and become pathogenic, which leads to the expression of the disease symptoms [7]. It is known that several factors may influence the grapevine susceptibility to GTDs, mainly climate, vine age, soil fertilization, rootstock, and cultivar and clones’ resistance [22,23,24].

Since it is known that these diseases are influenced by the type of pathogens involved, and their complete eradication is not possible, the identification, characterization, and early detection of the fungi involved are crucial to an early diagnosis and to the development of efficient control strategies [14,25,26]. Therefore, GTDs control is primarily focused on disease prevention and mitigation, based on an integrated management program, including physical, chemical, biological, and/or other control strategies [1,27].

In the past, the impact of GTDs was limited, applying sodium arsenate, which was then prohibited in Europe in 2003 due to its high toxicity on human health and environment [28]. Currently, treatments available to control GTDs are not totally efficient [1,17], which leads to the need to search for alternative methods against these diseases. The biological control, based on the use of endophytic microorganisms, has already shown potential and has been the focus of several studies [20,29,30,31]. The endophytes are defined as microorganisms that colonize healthy internal plant tissues without causing any apparent disease symptoms [32] and may also confer tolerance to environmental stresses and pathogens [33]. The endophytic community in a single plant is usually composed of numerous species of fungi [34], and their number and species composition are influenced by the environment, plant physiology, anthropogenic factors, and pathogen infections [30,35,36,37]. Therefore, for all the reasons listed before, the knowledge of endophytic communities in grapevine has an important role for future studies on disease management. Fungal endophytes have shown antagonistic effects against grapevine pathogens, such as fungi belonging to the genera *Alternaria* and *Epicoccum* against *Plasmopara viticola* and *Botrytis cinerea* [38,39]. Other endophytes belonging to the genera *Epicoccum*, *Cladosporium,* and *Alternaria* have revealed their potential as biological control agents against esca-associated fungi [40,41]. Besides, fungi like those belonging to the *Trichoderma* genus have demonstrated efficiency as biological control agents in grapevine. *Trichoderma* has shown protection of pruning wounds in nurseries [31], reducing the incidence of *Phaeomoniella chlamydospora* and *Phaeoacremonium* spp. in rootstock cuttings [42].

The necessity for new strategies against GTDs, the lack of information and data on grapevine endophytic communities in Portugal, especially in a major producing region, such as Alentejo, led to this study, which aimed to (1) identify the fungal endophytic community associated with important grapevine cultivars in Alentejo vineyards; (2) identify the phytopathogenic fungi associated with GTDs in the studied vineyards; (3) acquire new knowledge on the antagonistic interaction among some endophytes and GTDs phytopathogenic fungi.

## 2. Materials and Methods

### 2.1. Plant Material

Assays were carried out in September 2018 in three vineyards located in the Alentejo region (south of Portugal): site 1 (38°32′39.32″ N 7°52′10.6″ W), site 2 (39°03′00.3″ N 7°05′40.2″ W), and site 3 (38°26′42.2″ N 7°43′22.8″ W). Roots, petioles, and offshoots of asymptomatic and symptomatic plants were collected from the cultivars Trincadeira (from site 1 and site 2) and Alicante Bouschet (from site 3). Three asymptomatic and three symptomatic plants were sampled from sites 1 and 3, and five asymptomatic and five symptomatic plants were sampled from site 2.

From individual plants, eight branches were cut, and secondary roots (length 10–15 cm, diameter 3–5 mm) were collected from different parts of the root system. The root samples were placed into sterile plastic bags along a small portion of soil in order to preserve the humidity. All the samples were transported to the laboratory and stored at 4 °C until further analyses, which occurred within 48 h. Roots were separated from the soil by washing them in running water and treated with 0.05% (*v*/*v*) Tween 20 in 50 mL falcon tubes. Roots, petioles, and offshoots samples were decontaminated and treated, according to Spinosi et al. [28].

### 2.2. Fungal Isolation

After decontamination, plant pieces were dried in sterile Whatman paper, cut into sections, placed on Petri dishes of 90 mm diameter (five per plate) containing Potato Dextrose Agar medium (PDA, Merck, Darmstadt, Germany), and incubated for 1–2 weeks at 23–25 °C. Each of the different morphologically identified colonies was transferred through a small agar disk (about 5 mm^2^) of the growing fungus to a fresh 60 mm diameter PDA plate. The obtained colonies were grouped and numbered according to their morphological characteristics, based on shape, form, size, growth time, border, surface, opacity, pigmentation, and the shape and size of the fungal fruiting bodies, spores, and hyphae. Hyphae and spores were observed under an Olympus BX-5ed0 compound microscope (400× magnification).

### 2.3. Extraction of Genomic DNA (gDNA)

Once the entire dish surface was covered with the fungus, the mycelium was removed using a sterilized scalpel, and the gDNA was extracted following the CTAB method (hexadecyltrimethylammonium bromide) [43], with some modifications [30]. DNA concentration and quality were determined using a NanoDrop-2000C spectrophotometer (Thermo Scientific, Waltham, MA, USA). Samples were kept in the freezer (−20 °C) until further analyses.

### 2.4. Fungal DNA Identification

The internal transcribed spacer (ITS) region of nuclear rDNA was amplified through PCR from genomic DNA by using ITS1 and ITS4 primers [44]. PCR reactions consisted of 1 μL of gDNA, 1.5 mM MgCl_2_, 0.2 mM dNTPs (Fermentas, Thermo Scientific, Waltham, MA, USA), 1 mM of each primer, and 2.5 U of Dream-Taq DNA polymerase (Fermentas, Thermo Scientific, Waltham, MA, USA) in a total reaction volume of 50 mL. Amplification was carried out in a Thermal Cycler (Bio-Rad, Hercules, CA, USA) at 95 °C for 3 min, followed by 40 cycles of 95 °C for 30 s, 55 °C for 55 s, and 72 °C for 2 min and a final extension at 72 °C for 10 min. Amplified products were analyzed by 1% agarose gel electrophoresis prepared with 0.5 × Tris-Borate-EDTA buffer (TBE) and visualized on a Gene Flash Bio Imaging system (Syngene, Cambridge, UK). PCR reaction products were purified using the NZYGelpure kit (Nzytech, Lisbon, Portugal), according to the manufacturer’s protocol. Samples were quantified using a spectrophotometer NanoDrop-2000C (Thermo Scientific, Waltham, MA, USA) and sequenced in a sense strand by Macrogen Inc. (Madrid, Spain). ITS sequence homology was explored at the National Center for Biotechnology Information (NCBI) database using the BLAST algorithm (http://www.ncbi.nlm.nih.gov/) (BLASTn). All the fungal sequences that showed resemblance to at least 91% were used to identify the fungus analyzed.

### 2.5. Direct Inhibition Antagonism Test and Multivariate Data Analysis

The obtained fungal isolates were tested in vitro for their antagonistic activity using the direct opposition method [45], with some modifications. Briefly, a 3 mm mycelium plug from a pure culture of the potential antagonistic fungus actively growing was placed next to the edge of a 60 mm PDA dish, and, on the opposite side, a similar-sized mycelial plug of the GTDs pathogen was placed. For this study, six endophytic fungi described as potential antagonists were used [29,30,46,47,48,49]: *Fusarium oxysporum*, *Aspergillus niger*, *Penicillium* sp., *Trichoderma* sp., *Clonostachys rosea,* and *Epicoccum nigrum*, and three GTDs pathogens: *Diaporthe* sp., *Phialophora fastigiata,* and *Diplodia pseudoseriata*, totalizing 18 pathogen/antagonist combinations. Each combination of pathogen/antagonist was repeated 3 times as well as the controls, incubating the Petri dishes at 22 °C ± 2 °C, in dark conditions. During the incubation period, the radial growth towards the interacting fungus (internal radius) was measured daily by using a graduated ruler for nine days. Measurements were made from the edge of the plug with the mycelia of the pure culture until the end of the colony growth. The growth of the control fungus was also measured. The inhibition percentage (I) was calculated using the formula: I (%) = 100 × [(control − treatment)/control] [50].

The fungal growth values (radial growth in cm) were calculated using the fungal growth values dataset from each pathogenic fungus (*Diaporthe* sp., *D. pseudoseriata*, and *P. fastigiata*) in the presence of the potential antagonists (*F. oxysporum*, *A. niger*, *Penicillium* sp., *Trichoderma* sp., *C. rosea,* and *E. nigrum*). In addition, in each assay, the selected fungi were grown without any potential antagonists and values used as a control. A one-way permutational analysis of variance (PERMANOVA) was applied to test the hypothesis that significant differences existed between the growth values of the pathogenic fungi (*Diaporthe* sp., *D. pseudoseriata*, and *P. fastigiata*) in the presence of the different potential antagonists (*F. oxysporum*, *A. niger*, *Penicillium* sp., *Trichoderma* sp., *C. rosea,* and *E. nigrum*). The PERMANOVA analysis was carried out following the one-factor design: ‘Fungal growth’ (one level, fixed). Fungal growth values data were square-root transformed to scale down the importance of fungi with high growth and, therefore, increase the importance of the fungi with less growth in the analysis of the similarity between the fungi. The PERMANOVA analysis was conducted on a Bray– Curtis similarity matrix [51]. The null hypothesis was rejected at a significance level <0.05 (if the number of permutations was lower than 150, the Monte Carlo permutation *p* was used). Whenever significant interactions in the effects of the factor were detected, these were examined using posteriori pairwise comparisons, using 9999 permutations under a reduced model.

The statistical analyses were performed using the PRIMER v6 software [52] with the PERMANOVA add-on package [53].

## 3. Results

### 3.1. Fungal Isolation from Plant Material

From the 2681 organisms isolated, 1276 were obtained from asymptomatic plants, 1405 were obtained from symptomatic plants. Most of the isolates were obtained from offshoots (1082 isolates), followed by roots (820 isolates) and petioles (779 isolates) (Table 1). Based on morphological characteristics (shape and size of the fungal fruiting bodies, spores, and hyphae), 39 different fungi were identified, which were selected for molecular identification. Conventional PCR based on the ITS region, followed by sequencing and bioinformatic analysis, confirmed 39 fungi as different individuals, as shown in Table 1. Each isolate was identified as a taxonomic group at the species or genera level; 65% of the isolates were identified at the species level and 35% at the genera level.

The homologous identity score of the fungi performed in NCBI is listed in Table 1 and reveals a high degree of similarity with several fungi species or genera, with % of homology, which varies from 91% to 100%. Table 1 also indicates the number of isolates obtained from each fungus species or genera, according to GTDs symptomatology, in each plant organ and in the whole plant.

Through ITS sequencing together with microscopic analysis of morphological characteristics, it was possible to obtain a high diversity of endophytes—seven of them only obtained in asymptomatic plants (*Chaetomium succineum*, *Eupenicillium* sp., *Fusarium verticillioides*, *Penicillium glabrum*, *Pestalotiopsis* sp., *P. fastigiata*, *Phlebiopsis gigantea*), six only obtained in symptomatic plants (*Acrocalymma vagum*, *Aspergillus niveus*, *Aspergillus* sp., *Aspergillus terreus*, *Penicillium chrysogenum*, *Phialophora* sp.), and 26 obtained in both asymptomatic and symptomatic plants (*Alternaria alternata*, *Alternaria* sp., *Aspergillus niger*, *Beauveria bassiana*, *Bjerkandera adusta*, *B. cinerea*, *C. rosea*, *Clonostachys* sp., *Colletotrichum* sp., *Cytospora acaceae*, *Diaporthe* sp., *D. pseudoseriata*, *Epicoccum nigrum*, *F. oxysporum*, *Fusarium* sp., *Hormonema viticola*, *Macrophomina phaseolina*, *Neofusicoccum parvum*, *Penicillium* sp., *Penicillium thomii*, *Phlebia setulosa*, *Rutstroemiaceae* sp., *Stereum armeniacum*, *Talaromyces sp.*, *Trichoderma* sp., *Truncatella angustata*) (Table 1).

*Penicillium* sp. was the prevalent fungus obtained from the samples, with 702 isolates from asymptomatic and symptomatic plants, followed by *Trichoderma* sp. with 341 isolates, *F. oxysporum* with 182 isolates, *M. phaseolina* with 166 isolates, *A. niger* with 134 isolates, *H. viticola* with 126 isolates, *B. adusta* with 109, and *Diaporthe* sp. with 108. The remaining fungi showed less than 100 isolates (Table 1).

*Penicillium* sp. was also the prevalent fungus in both symptomatic and asymptomatic plants, with 406 isolates in asymptomatic plants and 296 in symptomatic plants, mainly obtained from offshoots but also isolated from roots and petioles. *Trichoderma* sp. isolates were also obtained in a substantially higher number in asymptomatic plants than in symptomatic plants, mainly from the roots. *M. phaseolina*, *A. niger,* and *P. glabrum* also showed a higher number of isolates in asymptomatic than in symptomatic plants. In opposition, the isolates of *B. cinerea*, *Diaporthe* sp., *F. oxysporum*, *Fusarium* sp., *H. viticola*, *N. parvum*, and *Rutstroemiaceae* sp. were present in a higher number in symptomatic plants than in asymptomatic plants. The other fungi identified presented less than 50 isolates in total (Table 1).

### 3.2. Grapevine Trunk Disease (GTDs)-Associated Fungi

Among the overall fungi that were identified, nine were previously described as GTDs pathogens, with six of them identified at the species level (*H. viticola*, *T. angustata*, *Stereum armeniacum*, *P. fastigiata*, *C. acaciae*, *D. pseudoseriata*) and three at the genera level (*Diaporthe* sp., *Pestalotiopsis* sp., *Neofusicoccum* sp.) (Table 1).

*Stereum armeniacum* is the only GTD-associated fungus belonging to Division Basidiomycota (Table 2). *D. pseudoseriata* and *N. parvum* belong to the Botryosphaeriaceae Family (Table 2). *T. angustata* and *Pestalotiopsis* sp. are both from Amphisphaeriaceae Family. *Diaporthe* sp. and *C. acaciae* are from Diaportales Order (Table 2).

Overall, almost all GTDs-associated fungi identified belong to Division Ascomycota (eight species/genera), represented by three classes, with the class Sordariomycetes being the most representative (five fungi), followed by the Dothideomycetes (two fungi) and the Eurotiomycetes (one fungus). Only one fungus was identified as belonging to Division Basidiomycota, represented by Class Agaricomycetes (Table 2).

*H. viticola* was the prevalent fungus in asymptomatic plants (53 isolates), followed by *N. parvum* (13 isolates) and *Diaporthe* sp. (11 isolates) (Table 1). The remaining fungi were represented by less than 10 isolates. In the symptomatic plants analyzed, the fungus with the highest number of isolates was *Diaporthe* sp. (97 isolates), followed by *H. viticola* (73 isolates), *N. parvum* (62), *C. acacia,* and *T. angustata* (11) (Table 1). Other identified fungi were represented by less than six isolates (Table 1). In general, more GTDs-associated fungal isolates from symptomatic plants were obtained.

*T. angustata* and *S. armeniacum* were identified only in roots in both symptomatic and asymptomatic plants. The presence of *H. viticola* was verified in all plant organs of asymptomatic and symptomatic plants, mainly in the petioles of asymptomatic plants with 28 isolates and in the roots of symptomatic plants with 29 isolates. The incidence of *Diaporthe* sp. was observed mainly in the offshoots of both asymptomatic and symptomatic plants. *N. parvum* was also verified in all plant organs, in asymptomatic and symptomatic plants, and its incidence was mainly observed in offshoots in symptomatic plants. *T. angustata* was only isolated from roots in both asymptomatic and symptomatic plants (Table 1).

### 3.3. Direct Inhibition Antagonism Tests

Direct inhibition antagonism tests were carried out using three GTDs-associated pathogens, chosen randomly, and six endophytic fungi, which were already reported to have antagonist activity against pathogens.

The fungal growth was observed and measured from the 1st to the 9th day for *Diaporthe* sp., *D. pseudoseriata*, and *P. fastigiata*. PERMANOVA statistical analyses were performed comparing the mycelial growth of the pathogens using the overall measurements from the 3rd, 6th, and 9th day of the tests.

#### 3.3.1. Evolution of the Control

By analyzing the growth of the control fungi over the days, it was verified that *P. fastigiata* was the only fungus to reach the edge of the Petri dish (Table 3). However, the mycelia of *D. pseudoseriata* and *E. nigrum* grew as fast as the mycelial growth of *P. fastigiata*. Despite the fact that *Diaporthe* sp. and *Penicillium* sp. showed mycelial growth only on the third day, *A. niger* showed less mycelial growth on day 9 within the fungi studied.

The growth of *Diaporthe* sp. started on the 3rd day (0.67 cm) and stopped on the 6th (3.80 cm). *P. fastigiata* started its growth on the 1st day (0.70 cm) and stopped it on the 5th day (4.10 cm), when the margins of the fungus reached the edge of the Petri dish. *D. pseudoseriata* started its growth on the 1st day (0.70 cm) and continued growing until the 4th day (4.00 cm). *F. oxysporum* started its growth on the 1st day (0.23 cm) and on the 9th day was still growing (2.80 cm). The growth of *A. niger* started on the 1st day and on the 9th reached 1.13 cm. *Penicillium* sp. had not started growing until the 3rd day (0.27 cm) and on the 9th reached 1.17 cm. *Trichoderma* sp. had growth from 1st (0.10 cm) to 9th day (1.78 cm). *C. rosea* started growing on the 2nd day (0.30 cm) and on the 9th day was still growing (2.30 cm). *E. nigrum* grew 0.43 cm on the first day and reached 4.03 cm on the 9th (Table 3).

#### 3.3.2. Antagonistic Action against *Diaporthe* sp.

**Figure 1:**
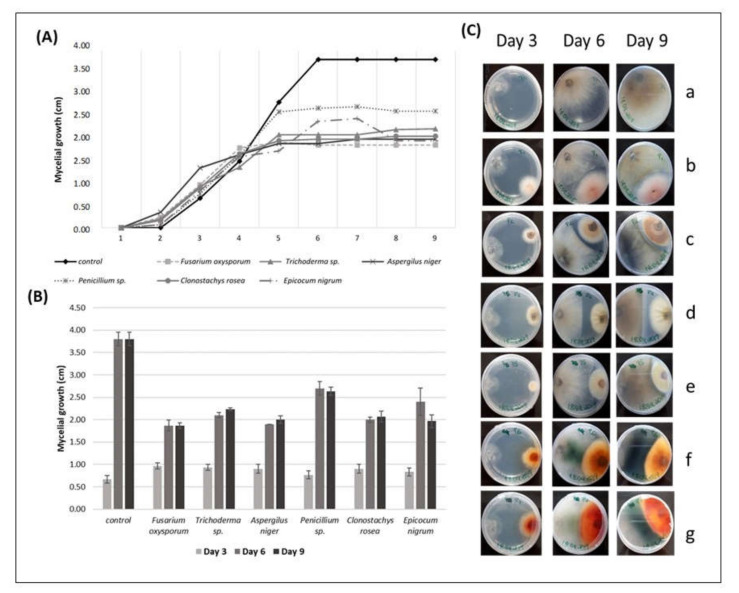
(**A**) The radial mycelial growth (cm) of *Diaporthe* sp. over time (days), registered in direct inhibition tests with some endophytic fungi; (**B**) radial growth values of *Diaporthe* sp. (cm) (r *Diaporthe* sp. ± SE) in the presence of different endophytic fungi on the 3rd, 6th, and 9th day of direct antagonism test; (**C**) interactions of *Diaporthe* sp., over the days, with grapevine endophytic fungi. *Diaporthe* sp. on the left side and the endophytic on the right side of the Petri dish. (**a**) *Diaporthe* sp. (control), (**b**) *Diaporthe* sp. vs. *Fusarium oxysporum*, (**c**) *Diaporthe* sp. vs. *Trichoderma* sp., (**d**) *Diaporthe* sp. vs. *Aspergillus niger*, (**e**) *Diaporthe* sp. vs. *Penicillium* sp., (**f**) *Diaporthe* sp. vs. *Clonostachys rosea*, (**g**) *Diaporthe* sp. vs. *Epicocum nigrum.* Pictures from the reverse side of the colonies.

Direct inhibition antagonism tests were carried out to verify the antagonist action of six endophytic fungi against the mycelial growth of *Diaporthe* sp. All the antagonists were able to inhibit the mycelial growth of the pathogen in the nine days of the direct inhibition test (Figure 1A–C). From the 5th day, there was a deceleration in the mycelial growth of the pathogens caused by the presence of the endophytic fungi when compared to the control. After the 6th day, all radial growth values of *Diaporthe* sp. were lower than the values of the control (Figure 1A), which was confirmed by statistical analyses (Figure 1B).

The PERMANOVA analysis revealed that the growth of *Diaporthe* sp. in control was always significantly higher (*p* < 0.01) compared to the *Diaporthe* sp. growth in the antagonism assay, showing that the mycelial growth of the pathogen did not reach the maximum when in the presence of endophytic fungi (Figure 1A,B). The average growth ± SE of *Diaporthe* sp. in the control dish ranged from 0.63 cm ± 0.09 on the 3rd day to 3.80 ± 0.15 on the 9th day.

The average mycelial growth of *Diaporthe* sp. on the 9th day of the test was 1.87 cm in the presence of *F. oxysporum*, which showed the highest antagonistic effect against the pathogen compared to the other fungi in the assay. The mycelial growth of *Diaporthe* sp. was higher in the presence of *Penicillium* sp. (2.63 cm) than in the presence of the other fungi, showing that *Penicillium* sp. presented the lowest antagonistic effect against the pathogen (Figure 1A). The PERMANOVA analysis also revealed that the mycelial growth of *Diaporthe* sp. in the presence of *Penicillium* sp. showed significantly higher values compared to *F. oxysporum* (*p <* 0.0052), *Trichoderma* sp. (*p* < 0.0162), *A. niger* (*p <* 0.0172), and *C. rosea* (*p <* 0.0177); however, no significant differences (*p* > 0.05) were found when compared to *E. nigrum*. The mycelial growth of *Diaporthe* sp., in the presence of *F. oxysporum*, showed no significant differences when compared to *A. niger*, *C. rosea,* and *E. nigrum* (*p* > 0.05) (Figure 1B).

The PERMANOVA analysis also revealed no significant differences in the mycelial growth of *Diaporthe* sp. in the presence of *Trichoderma* sp. compared to *A. niger*, *C. rosea,* and *E. nigrum* (*p* > 0.05), in the presence *A. niger* compared to *C. rosea* (*p* > 0.05), and in the presence of *C. rosea* compared to *E. nigrum* (Figure 1B, Table 4).

During interspecific mycelial interactions, all the endophytic fungi were able to stop the growth of the *Diaporthe* sp. once they were fighting for space (Figure 1C). Some of them, like *Trichoderma* sp. and *A. niger,* did not even touch the pathogen to stop its growth. *F. oxysporum*, *Penicillium* sp., *C. rosea,* and *E. nigrum* started touching the margins of the pathogen on the 5th day. Changes in the mycelium pigmentation of *Diaporthe* sp. were also observed during interspecific mycelial interactions. Margins of *Diaporthe* sp. colonies became lighter brown pigmented in the contact zone with *F. oxysporum* and dark green pigmented in the contact zone with *C. rosea* and *E. nigrum*, which was better observed on the reverse side of the plate (Figure 1C).

#### 3.3.3. Antagonism Action against *Diplodia pseudoseriata*

**Figure 2:**
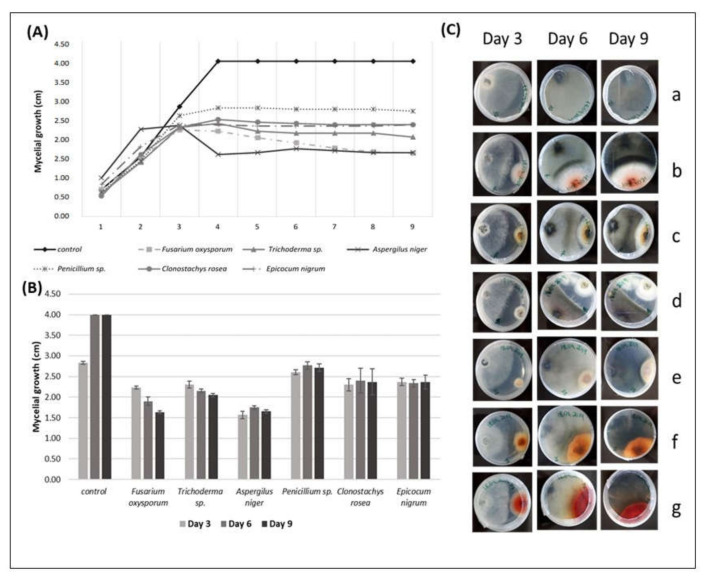
(**A**) The radial mycelial growth (cm) of *Diplodia pseudoseriata* over time (days), registered in direct inhibition tests with some endophytic fungi; (**B**) radial growth values of *Diplodia pseudoseriata* (cm) (r *D. pseudoseriata* ± SE) in the presence of different endophytic fungi on the 3rd, 6th, and 9th day of direct antagonism test; (**C**) interactions of *Diplodia pseudoseriata*, over the days, with grapevine endophytic fungi. *Diplodia pseudoseriata* on the left side and the endophytic on the right side of the Petri dish. (**a**) *Diplodia pseudoseriata* (control), (**b**) *Diplodia pseudoseriata* vs. *Fusarium oxysporum*, (**c**) *Diplodia pseudoseriata* vs. *Trichoderma* sp., (**d**) *Diplodia pseudoseriata* vs. *Aspergillus niger*, (**e**) *Diplodia pseudoseriata* vs. *Penicillium* sp., (**f**) *Diplodia pseudoseriata* vs. *Clonostachys rosea*, (**g**) *Diplodia pseudoseriata* vs. *Epicocum nigrum.* Pictures from the reverse side of the colonies.

By analyzing the mycelial growth of *D. pseudoseriata* over the days, it was verified that from the 3rd day, there was a deceleration in the pathogen growth caused by the presence of the endophytic fungi comparing to the control (Figure 2A–C). After the 4th day, all the growth radius values of *D. pseudoseriata* were lower than the radius values of the control (Figure 2A), which was confirmed by statistical analyses (Figure 2B).

The mycelial growth of *F. oxysporum* showed the highest antagonistic effect against *D. pseudoseriata* compared to the other fungi in the test, once the average mycelial growth of the pathogen on the 9th day of the test was 1.63 cm against the control’s average of 4.00 cm.

The mycelial growth of *D. pseudoseriata* was higher in the presence of *Penicillium* sp. (2.72 cm) than in the presence of the other fungi, confirming that *Penicillium* sp. showed the lowest antagonistic effect against this pathogen (Figure 2A).

The PERMANOVA analysis revealed that the mycelial growth of *D. pseudoseriata* in the presence of *F. oxysporum* showed significantly lower values compared to *A. niger* (*p* < 0.017), *E. nigrum* (*p* < 0.0123), and *Trichoderma* sp. (*p* < 0.0353); however, it showed no significant differences when compared to *C. rosea* (*p* > 0.05).

*Trichoderma* sp. in the presence of *D. pseudoseriata* showed significantly lower mycelial growth values compared to *A. niger* (*p* < 0.0137) and no significant differences compared to *C. rosea* (*p* > 0.05). The mycelial growth of *D. pseudoseriata* in the presence of *Penicillium* sp. showed significantly higher values compared to *F. oxysporum* (*p <* 0.0006), *Trichoderma* sp. (*p <* 0.0093), and *A. niger* (*p <* 0.0011); however, no significant differences were obtained when compared to *E. nigrum* (*p* > 0.05). *E. nigrum* showed significantly lower values of the mycelial growth of *D. pseudoseriata* compared to *A. niger* (*p <* 0.01) but showed no significant differences when compared to *Trichoderma* sp. and *C. rosea* (Figure 2B, Table 4).

The mycelial growth of *D. pseudoseriata* was stopped by all the endophytic fungi studied in the antagonism tests. However, the endophytic fungus *A. niger* did not even touch the pathogen to stop its growth. *E. nigrum* started touching the margins of the pathogen on the 3rd day; *F. oxysporum* and *Penicillium* sp. on the 4th day; *C. rosea* on the 5th day, and *Trichoderma* sp. on the 6th day. Changes in the mycelium pigmentation of *D. pseudoseriata* were also observed during interspecific mycelial interactions. The colony of *D. pseudoseriata* did not change color when interacting with *Penicillium* sp. In all the other interactions, the colonies of *Diplodia pseudoseriata* became dark brown (Figure 2C).

#### 3.3.4. Antagonism Action against *Phialophora fastigiata*

**Figure 3:**
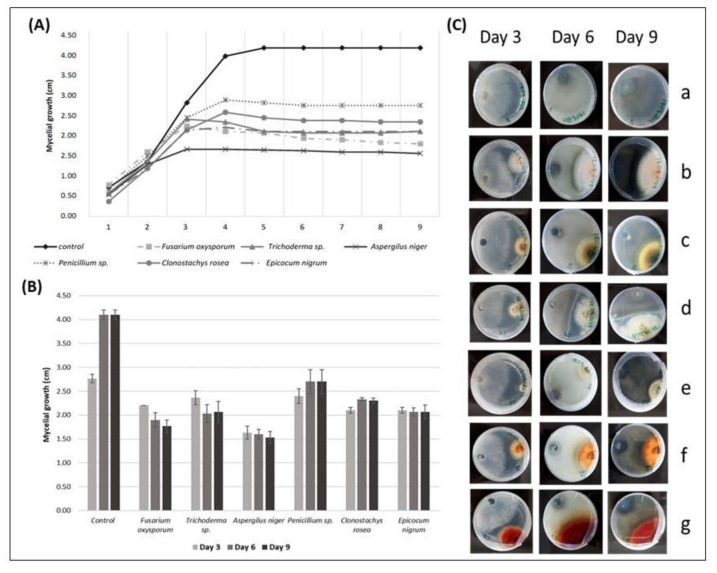
(**A**) The radial mycelial growth (cm) of *Phialophora fastigiata* over time (days), registered in direct inhibition tests with some endophytic fungi; (**B**) radial growth values of *P. fastigiata* (cm) (r *P. fastigiata* ± SE) in the presence of different endophytic fungi on the 3rd, 6th, and 9th day of direct antagonism test; (**C**) interactions of *P. fastigiata*, over the days, with grapevine endophytic fungi. *P. fastigiata* on the left side and the endophytic on the right side of the Petri dish. (**a**) *P. fastigiata* (control), (**b**) *P. fastigiata* vs. *Fusarium oxysporum*, (**c**) *P. fastigiata* vs. *Trichoderma* sp., (**d**) *P. fastigiata* vs. *Aspergillus niger*, (**e**) *P. fastigiata* vs. *Penicillium* sp., (**f**) *P. fastigiata* vs. *Clonostachys rosea*, (**g**) *P. fastigiata* vs. *Epicocum nigrum.* Pictures from the reverse side of the colonies.

By analyzing the mycelial growth of *P. fastigiata* over the days, a deceleration in the pathogen growth from the 3rd day was verified compared to the control, caused by the presence of the endophytic fungi. After the 4th day, all the radial growth of *P. fastigiata* was lower than the radius values of the control (Figure 3A). The PERMANOVA analysis revealed that the mycelial growth of *P. fastigiata* in control was always significantly higher (*p <* 0.01) compared to the *P. fastigiata* used in the antagonism assays (Figure 3B).

The average mycelial growth of *P. fastigiata* on the 9th day of the test was 1.53 cm in the presence of *A. niger*, which showed the highest antagonistic effect against the mycelial growth of the pathogen compared to the other fungi in the test. On the other hand, *Penicillium* sp. showed the lowest antagonistic effect in the growth of the mycelia of *P. fastigiata,* which reached 2.70 cm in the presence of this fungus (Figure 3A).

In addition, PERMANOVA analysis revealed that the mycelial growth of *P. fastigiata* in the presence of *Penicillium* sp. showed significantly higher values when compared to *F. oxysporum* (*p <* 0.0392) and to *A. niger* (*p <* 0.0087). The mycelial growth of *P. fastigiata* in the presence of *A. niger* showed significantly lower values when compared to *C. rosea* (*p <* 0.0059), *E. nigrum* (*p <* 0.0277), and *Trichoderma* sp. (*p <* 0.0381). *F. oxysporum* showed a significantly lower antagonistic effect compared to *C. rosea* (*p <* 0.0293).

The PERMANOVA analysis also revealed no significant differences between the remaining fungi (*p* > 0.05): *F. oxysporum* vs. *Trichoderma* sp., *F. oxysporum* vs. *A. niger*, *F. oxysporum* vs. *Penicillium* sp., *F. oxysporum* vs. *C. rosea*, *F. oxysporum* vs. *E. nigrum*, *Trichoderma* sp. vs. *A. niger*, *Trichoderma* sp. vs. *Penicillium* sp., *Trichoderma* sp. vs. *C. rosea*, *Trichoderma* sp. vs. *E. nigrum*, *Penicillium* sp. vs. *C. rosea*, *Penicillium* sp. vs. *E. nigrum,* and *C. rosea* vs. *E. nigrum* (Figure 3B).

The mycelial growth of *P. fastigiata* was stopped by all the endophytic fungi studied in the antagonism tests once they were fighting for space. In all interactions, the pathogen touched the margins of the endophytic fungi. All the endophytes started the mycelial growth on the first day; only *A. niger* mycelia did not start growing until the second day. *F. oxysporum* started touching the margins of the pathogen on the 3rd day; *Trichoderma* sp. and *E. nigrum* on the 4th day; *Penicillium* sp. and *C. rosea* on the 5th day, and *Trichoderma* sp. on the 8th day (Figure 3B, Table 4).

Changes in the mycelium pigmentation of *P. fastigiata* were observed during interspecific mycelial interactions. The pathogen did not change color only in the interaction with *Trichoderma* sp. In all the other interactions, the colonies of *P. fastigiata* became dark brown (Figure 3C).

#### 3.3.5. The Growth Inhibition of GTDs-Associated Fungi

The growth inhibition percentage of *Diaporthe* sp., *D. pseudoseriata,* and *P. fastigiata* was calculated on the 9th day of the direct inhibition test, using the formula described above. The inhibition percentages calculated for fungal isolates ranged from 30.70% to 62.60% (Table 5), showing that all endophytic fungi had some inhibitory action against the growth of the GTDs-associated fungi used in the tests. *Penicillium* sp. showed the lowest inhibition percentages values against the mycelial growth of *Diaporthe* sp., *D. pseudoseriata,* and *P. fastigiata*, ranging between 30.70% and 34.15% (Table 5), confirming the results described above. *F. oxysporum* showed the highest inhibition percentages values against *Diaporthe* sp. (50.88%) and *D. pseudoseriata* (59.17%), confirming the results already described. *A. niger* presented the highest antagonistic effect against *P. fastigiata,* with inhibition percentages of 62.60% (Table 5).

## 4. Discussion

### 4.1. Fungi Identification

Samples were collected from three different parts of plants (roots, petioles, and outshoots) showing GTDs symptoms (symptomatic plants) and from plants without GTDs symptoms (asymptomatic plants). The chosen cultivars for the study are important and representative cultivars for wine production from the Alentejo region as Trincadeira and Alicante Bouschet. These cultivars are included in the list of the ones allowed for wine production with the European nomenclature of Protected Designation of Origin (PDO), with several studies performed on their susceptibility to different pathogens and resistance to diseases.

In total, 2681 organisms were isolated from the samples, with fungal-like morphological characteristics. The fungi isolated were grouped based on morphological characteristics, which allowed the identification of 39 different molecular different individuals.

Despite the fact that the ITS region has a low taxonomic resolution for some species delimitations, mainly in some highly speciose genera, such as *Aspergillus*, *Cladosporium*, *Fusarium*, *Penicillium,* and *Trichoderma* [54,55], it is the main genetic marker for molecular identification at the species level and can be very suitable in some situations [56], as in the present study, where 67.5% of the individuals were identified at the species level, which was enough for the study proposes. Moreover, the molecular characterization through the ITS region corroborated the results achieved with the morphological identification.

#### 4.1.1. Endophytes Fungal Community

Most of the isolates were obtained from offshoots (1082 isolates), followed by roots (820 isolates) and petioles (779 isolates). From the total organisms isolated, 1276 were obtained from asymptomatic plants, 1405 were obtained from symptomatic plants. In a total of 39 different endophytic fungi identified, 26 were obtained from both asymptomatic and symptomatic plants, seven of them were only obtained from asymptomatic plants (*C. succineum*, *Eupenicillium* sp., *F. verticillioides*, *P. glabrum*, *Pestalotiopsis* sp., *P. fastigiata*, *P. gigantea*), and six were only obtained from symptomatic plants (*A. vagum*, *A. niveus*, *Aspergillus* sp., *A. terreus*, *P. chrysogenum*, *Phialophora* sp.).

The identification of endophytic fungal community is essential to determine the endophyte distribution in the vineyard to understand the role of the communities on the host and their influence in wine characteristics, for example, and to analyze their relationship with plant pathogens or diseases [57]. Most endophytes reside in the plant tissues without causing any effect on the host. Some of them can have mutualistic relationships with plants; others can provide benefits to their host through the promotion of plant growth and biocontrol against plant pathogens. Nevertheless, when environmental conditions become favorable, some endophytes may also exhibit pathogenic activity [58]. The importance to identify the endophytic fungal communities in the grapevines comes not only to identify some fungi in the plant, which can interfere in the wine quality through the production of toxic metabolites, such as fungi belonging to *Aspergillus* and *Penicillium* genera [30], but also to understand their relationships with known pathogens, so that control strategies may be developed.

Fungi belonging to *Fusarium*, *Penicillium*, *Alternaria,* and *Botryosphaeria* genera are referred to as the dominant endophytic fungi found in grapevine plants [59]. In this study, *Penicillium* sp. was the prevalent fungus, with 702 isolates—406 isolates obtained from asymptomatic plants and 296 from symptomatic plants. *Penicillium* sp. were mainly isolated from offshoots; however, they were also isolated from roots and petioles. Fungi belonging to *Penicillium* genera are the most common fungi reported in soil, air, and plants, playing the role of saprophytes or endophytes [60].

Limited studies can be found on the grapevine endophytic microbial community, with most researches focusing on bacterial endophytes [61]. In Alentejo vineyards, an endophytic community study was already performed by Varanda et al. [30], who identified 240 isolates belonging to 16 operational taxonomical units (OTUs), which is substantially less in number and in the variability of the endophytic community than the isolates obtained in the present work. Nevertheless, it is important to consider that different factors can shape the grapevine microbiome, such as seasonality, plant genotype, age, pedo-climatic features, surrounding wild plants, presence of a pathogen, the variety of the host plant, and others [57].

#### 4.1.2. GTDs-Associated Fungi

In the study presented here, among the 39 fungi identified morphologically and by molecular techniques, nine were associated with GTDs, with six identified at the species level (*H. viticola*, *T. angustata*, *S. armeniacum*, *P. fastigiata*, *C. acaciae*, *D. pseudoseriata*) and three at genera level (*Diaporthe* sp., *Pestalotiopsis* sp., *Neofusicoccum* sp.).

GTDs are primarily caused by ascomycetous fungi. However, some basideomiceteous taxa are also thought to play an important role in this disease complex [1,62], such as *S. armeniacum* from the *Stereaceae* family, the only fungus belonging to the *Basidiomycota* Division identified in this study. Among the ascomycetous identified in this work, two belong to the *Botryosphaeriaceae* family, two to *Dothioraceae*, two to *Amphisphaeriaceae*, one to *Herpotrichiellaceae,* and one to *Valsaceae*.

Fungi from the *Botryosphaeriaceae* family can cause cankers and consequent dieback in the most important grape growing areas in the world and are associated with the disease “Botryosphaeria dieback” of grapevines [63]. *Diplodia pseudoseriata* and *N. parvum*, identified in this work, are two of the most frequently isolated *Botryosphaeriaceae* fungi in grapevine areas worldwide [25].

*H. viticola* and *Diaporthe* sp. belong to the *Dothioraceae* family. *H. viticola* is a new fungus associated with GTDs, and it was first identified in the Canary Islands from the grapes in *Vitis vinifera* cv. Malvasia [64]. The generic names *Diaporthe* and *Phomopsis* are no longer used to distinguish different morphs of this genus, and *Diaporthe* is the denomination used nowadays [65,66]. Pathogenicity of several *Phomopsis spp./Diaporthe spp*., including *Phomopsis viticola* (Ascomycota, *Diaporthales*; syn. = *Diaporthe ampelina*), on grapevines has been well-established on green shoots of new vegetative growth [67]; however, it is now clear that *Phomopsis viticola* can be also associated with cankers in addition to the green parts of the grapevines [63], and this fungus is the principal pathogen associated with “Phomopsis dieback disease” [68].

*T. angustata* and *Pestalotiopsis* sp., commonly known as pestalotioid, belong to the *Amphisphaeriaceae* family and have been reported from grapevines with decline symptoms [69]. *Pestalotiopsis* sp. and *Truncatella* sp. are associated with grapevine cankers in Texas. The pathogenicity of a *Truncatella* sp. has shown low virulence and low percentage recovery from necrotic tissue, indicating that this species may act as a weak and/or opportunistic pathogen on grapevine [63]. *P. fastigiata*, originally described as *Cadophora fastigiata* [70], belongs to the Herpotrichiellaceae family, and it is associated with the decline of grapevines and has been reported from many grapevines growing countries, causing wood lesions and black streaking in longitudinal stem sections, the typical internal symptoms of esca and Petri disease [71]. *C. acaciae* belongs to the genus *Cytospora*, the family Valsacea. *Cytospora* sp. canker shows some of the same general dieback-type symptoms as botryosphaeria, eutypa, and phomopsis diebacks, placing these fungi in the GTDs complex [72].

In the present study, it was possible to identify GTDs pathogens in symptomatic and asymptomatic plants. Environmental conditions can activate virulence factors, leading to the pathogenicity of fungi due to the alteration of plant-fungus balance [73], resulting in disease in the host. Plant diseases may result from continuous irritation generated by a pathogen, which causes malfunction of host cells and their tissues [74], and it leads to the development of symptoms. However, if the environmental conditions are not favorable, and the pathogenicity of the fungus is not activated, the fungus can enter a latent state and remain inside the host without causing any symptoms [75]. The presence of antagonistic microorganisms can also difficult the development of the disease, stopping the colonization of the pathogen by the competition for nutrition and space or by the production of secondary metabolites that inhibit fungal growth [29,76].

In this study, the most commonly identified fungus associated with GTDs in asymptomatic plants was *H. viticola*, present in all plant tissues. However, the highest incidence of this fungus in asymptomatic plants was observed in the petioles of the plants. The incidence of fungi belonging to the *Diaporthe* genera in asymptomatic plants was not high; however, it was possible to identify them in the three areas, in the two cultivars, and in all plant organs studied. *Diaporthe* spp. comprises pathogenic, endophytic, and saprophyte species [77]; therefore, fungi from this genus are frequently present in asymptomatic plant tissues [78]. Even though there is a lack of information about *H. viticola*, especially linked to grapevine plants, this fungus belongs to the same family as *Diaporthe*, which can be an interesting beginning for future studies. *Neofusicoccum* species, *T. angustata*, *S. armeniacum*, *C. acaciae* were verified in asymptomatic plants. Once inside the host, endophytes enter a latent state [75], which can be the main reason for the late onset of symptoms caused by the infection of GTDs pathogens. Plant-endophyte balance can be altered by environmental conditions, which can activate virulence factors, leading to the pathogenicity of the fungus [73], which explains the presence of phytopathogenic fungi, identified also inside asymptomatic plants [30].

In symptomatic plants, the predominant GTDs-associated fungi in the three areas studied were *Diaporthe* sp., *H. viticola,* and the fungi associated with *Neofusicoccum* genera. *Phomopsis viticola* is also one of the predominant species identified in a field study in several vineyards in Portugal [79]. The fungus *P. fastigiata* was identified in symptomatic plants of Trincadeira cultivar. *T. angustata*, *S. armeniacum*, *C. acaciae*, *D. pseudoseriata,* and the fungi from *Pestalotiopsis* genera were identified on only a few occasions.

GTDs pathogens were verified in all plant organs; however, their incidence in the organs showed differences since vascular fungi like GTDs pathogens do not colonize systemically [80]. In other words, once inside the plant, vascular fungi responsible for cankers colonize the organ, which is infected, and their spores do not spread throughout the plant. *Diaporthe* sp. was the only fungus whose incidence was observed similarly in asymptomatic and symptomatic plants, with the offshoot the most infected organ, followed by petiole and root, respectively, showing that the infection of the pathogen in the plant could possibly have happened mainly through pruning wounds [1,18,80]. Even though GTDs-associated fungi infections happen primarily by pruning wound, the infection by those pathogens can also happen through any type of open wounds, such as those caused by retraining, trimming, and de-suckering [81], which can possibly explain the identification of GTDs-associated fungi in different organs of the plants. Some GTDs pathogens, like fungi responsible for black foot, are soilborne and are commonly found in nursery fields and soils; therefore, inoculum may already exist in soils before plantation, and infection can happen by some wounds caused by culture management [1], which explains the incidence of some phytopathogens in roots, such as *T. angustata* and *S. armeniacum*, verified only in the roots of both asymptomatic and symptomatic plants.

### 4.2. Endophyte Antagonism Activity

To provide efficient control against GTDs, researchers and specialists have been waging in control techniques set, which can involve also biological alternatives. Biological control against fungi has been studied in the last decades, and some researchers believe that it can be a good alternative to maintain GTDs-associated fungi under control. Several studies have shown that some fungal endophytes have beneficial effects on their hosts, such as in grapevines, showing antagonistic properties against some important pathogens [30]. Antagonist microorganisms can be used as biological control agents, contributing to achieving productive and sustainable agriculture. Thus, the investigation has been conducted on the diversity, distribution, and influence of endophytic fungi on the development and/or prevention of certain fungal diseases [46].

With the present work, we aimed to identify and study the GTDs-associated fungi with the intention of contributing to alternatives for reducing the incidence of these diseases. Thus, a better understanding of the role of endophytes in this GTDs complex was pertinent. The wide variety of endophytic fungi identified in this research led to a study of the interaction between possible antagonistic endophytes and GTDs phytopathogenic-associated fungi, as a starting point for the development of a biological control method. Therefore, direct inhibition antagonism was tested in vitro, using fungi identified in this research: three GTDs phytopathogenic (*Diaporthe* sp., *P. fastigiata,* and *D. pseudoseriata)* and six potential antagonistic endophytes (*F. oxysporum*, *A. niger*, *Penicillium* sp., *Trichoderma* sp., *C. rosea,* and *E. nigrum*).

All the endophytes used for the direct inhibition antagonism tests were able to negatively affect the growth of the GTDs-associated fungi, showing that non-pathogenic microorganisms can possibly protect hosts through their competition with phytopathogen for space and nutritional resources. However, it is not possible to confirm that the competition is the only mechanism responsible for the pathogen growth inhibition, once the antagonistic properties of biocontrol agents are based on the activation of multiple mechanisms, such as the competition for nutrients and space, mycoparasitism, antibiosis, metabolite production, or volatile compounds production [29,46,82].

*A. niger* was the only fungus that did not even touch the mycelium of the pathogen *Diaporthe* sp. and *D. pseudoseriata;* nevertheless, it still stopped the mycelium growth of those pathogens. The growth-inhibitory effects before physical contact between fungi may suggest the antagonistic mode of action that can also occur due to the production of certain metabolites by endophytic fungi, rather than just competition or parasitism [83].

On the 9th day of the test, inhibition percentages were calculated based on the control of each phytopathogen. *F. oxysporum* showed the highest percentage inhibition rate on the mycelial growth of *Diaporthe* sp. and *D. pseudoseriata*, and it presented the inhibition rates above 50% of the mycelial growth of *P. fastigiata*. In this study, the number of isolates of *F. oxysporum* obtained from symptomatic plants (145 isolates) was higher than obtained from asymptomatic (37 isolates). Although the number of *F. oxysporum* isolates increased, this fungus did not seem to help to inhibit the increase of the growth of *Diaporthe* sp. on symptomatic (97 isolates) compared to asymptomatic plants (11 isolates). *Fusarium* species have been reported to have antagonistic activity against *Colletotrichum acutatum* in olives trees [29]. However, some strains of *F. oxysporum* are known to be soil pathogens and responsible for grapevine decline and death [84,85].

Many antagonistic microorganisms have been proved to be active in vitro or in vivo. Among this list, the most well-known are the fungi from genera *Trichoderma*, *Aspergillus,* and *Penicillium* [48]. *A. niger* was the most potent inhibitor for the mycelium growth of *P. fastigiata* (62.60%), even if its mycelia only started growing on the second day, and it showed a slow mycelial growth over the days. However, in this study, the number of *A. niger* isolates from asymptomatic plants (79) was higher than from symptomatic plants (55). The success of antagonistic activity of *A. niger* has been confirmed in a study with *Colletotrichum acutatum* from olive trees, and the antagonistic efficiency has been assigned to the rapid growth and competition for space/nutrients, which is the opposite result showed in the study mentioned in the sentence below [29].

Although *Penicillium* spp. was the most abundant fungus in the present work, it demonstrated to be the least efficient antagonism fungus against the growth of the three GTDs pathogens, with inhibition rates between 30% and 35%. The number of *Penicillium* sp. isolates was higher in asymptomatic plants (406 isolates) than in symptomatic (296 isolates), which could be affected by the growth of other fungi or environmental conditions. However, fungi belonging to this species are considered to have interesting antagonistic activity against diverse pathogenic fungi due to the production of secondary metabolites with antibiotic activity [46]. They have the capacity to produce bioactive secondary metabolites, which has been stimulating researchers to investigate their involvement in plant protection based on a possible induction of antagonistic effects toward plant pathogens [61]. Many *Penicillium* species have been reported having antagonistic effects against plant pathogens using the induction of resistance, the production of antibiotic compounds, and the establishment of mycoparasitic interactions as mechanisms of action [86].

*Trichoderma* spp. are the most widely studied biological control agents for root and shoot pathogens to combat a wide range of plant diseases [87]. In this study, *Trichoderma* sp. was the second most abundant fungus, and it showed an effective effect in the pathogen’s growth inhibition, with mycelial inhibition rates under 50% in all tests. As expected, the number of *Trichoderma* sp. isolates was higher in asymptomatic plants (263 isolates) than in symptomatic (78 isolates), which could affect the growth of other fungi, such as GTDs pathogens. *Trichoderma*-based biocontrol mechanisms are mainly relying on mycoparasitism, production of antibiotic and/or hydrolytic enzymes, competition for nutrients, as well as induced plant resistance and numerous secondary metabolites, which can act directly or indirectly against the targeted pathogens of cultivated and forestry plants [49,88].

*C. rosea* and *E. nigrum* presented inhibition percentages around 40% and 50% in the mycelia growth of all pathogens studied. Both fungi showed a higher number of isolates in symptomatic plants than in asymptomatic. *Epicoccum* species have also shown antagonism activity against some grapevine phytopathogens like *P. viticola* and *B. cinerea* [30]. *E. nigrum* has been studied as a promising biocontrol agent and has been developed commercially due to its capability to produce secondary metabolites with antibiotic activity [89]. *C. rosea* has already been used as a biological agent in some crops. This biocontrol agent acts by two forms of antagonism: parasitism of hypha and competition for space and nutrition. During parasitism, the antagonistic fungus can remove pathogenic hyphae from the substrate and the previously colonized tissues [47].

Changes in pathogen mycelium pigmentation were observed during almost all interspecific mycelial interactions. *Diaporthe* sp. changed mycelium pigmentation from light brown to almost black when in contact with *Trichoderma* sp. *Diaporthe* sp. mycelium also changed pigmentation from light brown to dark-green when in contact with *C. rosea* and *E. nigrum*. The mycelium pigmentation of *D. pseudoseriata* only did not change when interacting with *Penicillium* sp. In all the other interactions, the colonies of *D. pseudoseriata* changed from light to dark brown. In the interaction between *P. fastigiata* and the endophyte, only *Trichoderma* sp. did not affect the pathogen’s mycelial pigmentation. The colors of the *P. fastigiata* colonies changed to dark brown in the interaction with the other endophytes. During interspecific mycelial interactions, changes in mycelium pigmentation of *Colletotrichum acutatum* in the contact zone with *E. nigrum*, *Aspergillus brasiliensis,* and *Aspergillus* sp. colonies were observed. The formation of pigments in the fungi mycelium can be a mechanism of the pathogen to protect hyphae from the antagonistic fungi by preventing access by cell wall degrading enzymes [90].

## 5. Conclusions

Grapevine endophytic community can have a wide range of fungi diversity, as shown in the present study using some GTDs’ asymptomatic and symptomatic grapevines in the Alentejo region. It is important to reinforce the idea that we are considering endophytes as fungi that reside in the plant tissues without causing any effect on the host. Nevertheless, when the environmental conditions become favorable to them, some endophytes may also exhibit pathogenic activity. *Penicillium* sp. and *Trichoderma* sp. were the fungi with the highest number of isolates, showing more isolates in asymptomatic than in symptomatic plants. This strongly agrees with their antagonistic role, protecting plants against pathogenic fungi.

Endophyte fungi were identified in roots, petioles, and offshoots, with offshoots presenting the highest number of isolates. Among the fungi identified, nine were associated with GTDs phytopathogens. It was verified that *Diaporthe* sp., *Neofusicoccum* sp., and *H. viticola* were the prevalent GTDs-associated fungi in symptomatic plants, although they were also identified in asymptomatic plants. *H. viticola* was the prevalent fungus verified in the samples, mainly in asymptomatic plants; however, its role in the GTDs complex has not been much explored yet. The presence of GTDs-associated fungi in asymptomatic plants corroborates the statements that they can also survive, part of their lives, as endophytes without causing any symptoms to the host, which can also explain the long latency time of the diseases. The great incidence of GTDs-associated fungi in different organs of the plants (roots, petioles, and offshoots) demonstrates that those pathogens are easily spread in the plant and in the vineyard.

Furthermore, some fungal genus/species with characteristics of biological antagonists were identified, and their antagonist activity was verified through direct inhibition. All endophytic fungi tested in the endophyte/pathogen interaction presented the inhibition of pathogens’ mycelia growth, verified by competition for nutrients and space. *F. oxysporum* was the most effective antagonist against *Diaporthe* sp. and *D. pseudoseriata*, but it also showed an antagonistic effect against *P. fastigiata*. *A. niger* showed to be the most effective inhibitor of the growth of *P. fastigiata*. *Penicillium* sp. showed to be the less effective inhibitor against the mycelial growth of the three GTDs-associated fungi studied.

Despite the fact that the endophyte community and their role in the GTDs in the Alentejo region still need further studies, with the results here presented, we reinforce the idea that plant hosts are important sources of potential biocontrol agents against GTDs-associated fungi, and their presence seems to hold the development of GTDs symptoms in affected plants.

## Figures and Tables

**Table 1:** Fungi identified morphologically and molecularly through ITS region, collected in vineyards located in the Alentejo region. The percentage of sequence homology explored at the NCBI database using the BLAST algorithm and the respective number of isolates according to symptomatology and plant organs are indicated.

		Roots		Petioles		Offshoots		Total	
	Homology	Asymptomatic Plants	Symptomatic Plants	Asymptomatic Plants	Symptomatic Plants	Asymptomatic Plants	Symptomatic Plants	Asymptomatic Plants	Symptomatic Plants
*Acrocalymma vagum*	100%	0	3	0	0	0	0	0	3
*Alternaria alternata*	100%	2	19	0	14	0	0	2	33
*Alternaria* sp.	100%	21	21	0	8	0	0	21	29
*Aspergillus niger*	99.47%	29	16	28	16	22	23	79	55
*Aspergillus niveus*	100%	0	0	0	1	0	0	0	1
*Aspergillus* sp.	98.75%	0	0	0	1	0	0	0	1
*Aspergillus terreus*	100%	0	1	0	0	0	0	0	1
*Beauveria bassiana*	98.65%	5	7	5	0	0	0	10	7
*Bjerkandera adusta*	99.14%	14	20	13	14	23	25	50	59
*Botrytis cinerea*	99.50%	9	26	0	6	1	23	10	55
*Chaetomium succineum*	99.71%	3	0	0	0	0	0	3	0
*Clonostachys rosea*	100%	11	7	0	4	7	23	18	34
*Clonostachys* sp.	100%	13	0	0	0	0	11	13	11
*Colletotrichum* sp.	100%	1	4	19	16	0	0	20	20
*Cytospora acaceae*	97.89%	0	0	6	0	3	11	9	11
*Diaporthe* sp.	99.66%	1	15	4	35	6	47	11	97
*Diplodia pseudoseriata*	100%	0	0	0	0	1	1	1	1
*Epicoccum nigurum*	100%	2	15	7	0	0	1	9	16
*Eupenicillium* sp.	97.89%	4	0	0	0	0	0	4	0
*Fusarium oxysporum*	99.71%	21	42	6	50	10	53	37	145
*Fusarium* sp.	95.25%	0	10	1	14	10	38	11	62
*Fusarium verticillioides*	99.72%	1	0	0	0	1	0	2	0
*Hormonema viticola*	92.05%	19	29	28	19	6	25	53	73
*Macrophomina phaseolina*	100%	44	19	28	15	30	30	102	64
*Neofusicoccum parvum*	98.02%	3	13	5	23	5	26	13	62
*Penicillium chrysogenum*	100%	0	4	0	0	0	0	0	4
*Penicillium glabrum*	91.09%	12	0	25	0	31	0	68	0
*Penicillium* sp.	99.27%	59	32	76	98	271	166	406	296
*Penicillium thomii*	100%	17	33	8	12	0	0	25	45
*Pestalotiopsis* sp.	99.72%	0	0	0	0	1	0	1	0
*Phialophora fastigiata*	99.75%	1	0	0	0	0	0	1	0
*Phialophora* sp.	99.50%	0	2	0	0	0	11	0	13
*Phlebia setulosa*	96.60%	2	21	0	0	0	0	2	21
*Phlebiopsis gigantea*	99.08%	4	0	0	0	0	0	4	0
*Rutstroemiaceae* sp.	100%	7	0	5	44	2	40	14	84
*Stereum armeniacum*	100%	4	5	0	0	0	0	4	5
*Talaromyces* sp.	100%	1	8	0	0	0	0	1	8
*Trichoderma* sp.	99.47%	108	10	88	37	67	31	263	78
*Truncatella angustata*	100%	9	11	0	0	0	0	9	11
**Total**		**427**	**393**	**352**	**427**	**497**	**585**	**1276**	**1405**

ITS: Internal Transcribed Spacer; NCBI: National Center for Biotechnology Information; BLAST: Basic Local Alignment Search Tool

**Table 2:** Taxonomy of the GTDs-associated fungi identified in vineyards located in the Alentejo region.

Species/Genus	Genus	Family	Order	Class	Division
*Hormonema viticola*	Hormonema	Dothioraceae	Diaporthales	Sordariomycetes	Ascomycota
*Truncatella angustata*	Truncatella	Amphisphaeriaceae	Xylariales	Sordariomycetes	Ascomycota
*Stereum armeniacum*	Stereum	Stereaceae	Russulales	Agaricomycetes	Basidiomycota
*Phialophora fastigiata*	Phialophora	Herpotrichiellaceae	Chaetothyriomycetidae	Eurotiomycetes	Ascomycota
*Cytospora acaciae*	Cytospora	Valsaceae	Diaporthales	Sordariomycetes	Ascomycota
*Diplodia pseudoseriata*	Diplodia	Botryosphaeriaceae	Botryosphaeriales	Dothideormycetes	Ascomycota
*Diaporthe* sp.	Diaporthe	Diaporthaceae	Diaporthales	Sordariomycetes	Ascomycota
*Pestalotiopsis* sp.	Pestalotiopsis	Amphisphaeriaceae	Xylariales	Sordariomycetes	Ascomycota
*Neofusicoccum* sp.	Neofusicoccum	Botryosphaeriaceae	Botryosphaeriales	Dothideormycetes	Ascomycota

GTDs: grapevine trunk diseases.

**Table 3:** Growth of the control fungi (in cm) over the nine days of the tests (mean values from three replicates).

Fungus	Day								
	1	2	3	4	5	6	7	8	9
*Diaporthe* sp.	0	0	0.67	1.50	2.83	3.80	3.80	3.80	3.80
*Diplodia pseudoseriata*	0.70	1.53	2.83	4.00	4.00	4.00	4.00	4.00	4.00
*Phialophora fastigiata*	0.70	1.33	2.77	3.90	4.10	4.10	4.10	4.10	4.10
*Fusarium oxysporum*	0.23	0.50	0.90	1.17	1.53	1.77	2.13	2.37	2.80
*Aspergillus niger*	0.03	0.20	0.37	0.57	0.73	0.87	0.97	0.97	1.13
*Penicillium* sp.	0	0	0.27	0.53	0.90	1.00	1.07	1.10	1.17
*Trichoderma* sp.	0.10	0.30	0.60	0.87	1.07	1.20	1.40	1.68	1.78
*Clonostachys rosea*	0	0.30	0.80	1.07	1.57	1.67	1.97	2.05	2.30
*Epicocum nigrum*	0.43	0.70	1.23	1.70	2.20	2.73	3.17	3.70	4.03

**Table 4:** Inhibition of *Diaporthe* sp., *Diplodia pseudoseriata*, and *Phialophora fastigiata* by the endophytic fungus *Fusarium oxysporum*, *Trichoderma* sp., *Aspergillus niger*, *Penicillium* sp., *Clonostachys rosea*, and *Epicocum nigrum*. *p*-values of the one-factor PERMANOVA analysis. Bold values highlight significant differences (*p* < 0.05).

	*Diaporthe* sp.	*D. pseudoseriata*	*P. fastigiata*
		*p*-Values	
*F. oxysporum* vs. Control	**0.0002**	**0.0001**	**0.0005**
*Trichoderma* sp. vs. Control	**0.0002**	**0.0002**	**0.0026**
*A. niger* vs. Control	**0.0014**	**0.0003**	**0.0003**
*Penicillium* sp. vs. Control	**0.0077**	**0.0005**	**0.0131**
*C. rosea* vs. Control	**0.0011**	**0.0126**	**0.0003**
*E. nigrum* vs. Control	**0.005**	**0.0008**	**0.0003**
*F.oxysporum* vs. *Trichoderma sp.*	**0.0485**	**0.0353**	0.386
*F. oxysporum* vs. *A. niger*	0.5766	**0.017**	0.0566
*F. oxysporum* vs. *Penicillium sp.*	**0.0052**	**0.0006**	**0.0392**
*F. oxysporum* vs. *C. rosea*	0.3764	0.1104	**0.0293**
*F. oxysporum* vs. *E. nigrum*	0.267	**0.0123**	0.244
*Trichoderma sp.* vs. *A. niger*	0.1731	**0.0137**	**0.0381**
*Trichoderma sp.* vs. *Penicillium sp.*	**0.0162**	**0.0093**	0.1309
*Trichoderma sp.* vs. *C. rosea*	0.3709	0.6528	0.2162
*Trichoderma sp.* vs. *E. nigrum*	0.6012	0.2782	0.5927
*A. niger* vs. *Penicillium sp.*	**0.0172**	**0.0011**	**0.0087**
*A. niger* vs. *C. rosea*	0.7164	0.0803	**0.0059**
*A. niger* vs. *E. nigrum*	0.5322	**0.01**	**0.0277**
*Penicillium* sp. vs. *C. rosea*	**0.0177**	0.2489	0.1617
*Penicillium* sp. vs. *E. nigrum*	0.1266	0.0583	0.0757
*C. rosea* vs. *E. nigrum*	0.781	0.9857	0.1702

**Table 5:** Inhibition percentages of *Diaporthe* sp., *Diplodia pseudoseriata*, and *Phialophora fastigiata* by the endophytic fungus *Fusarium oxysporum*, *Trichoderma* sp., *Aspergillus niger*, *Penicillium* sp., *Clonostachys rosea*, and *Epicocum nigrum*, calculated 9 days after inoculation.

	Inhibition Percentage		
	*Diaporthe* sp.	*D. pseudoseriata*	*P. fastigiata*
*F. oxysporum*	50.88%	59.17%	56.91%
*Trichoderma* sp.	41.23%	48.75%	49.59%
*A. niger*	47.37%	58.75%	62.60%
*Penicillium* sp.	30.70%	32.08%	34.15%
*C. rosea*	45.61%	40.83%	43.90%
*E. nigrum*	48.25%	40.83%	49.59%
